# Supplementary material for: Empirical methods for controlling false positives and estimating confidence in ChIP-Seq peaks
Source: BMC Bioinformatics. 2008 Dec 5;9:523. doi: 10.1186/1471-2105-9-523 (PMC2628906; doi:10.1186/1471-2105-9-523)
Supplement: Additional File 1 — A variety of html documents from the USeq web site detailing the available applications, their best usage, output file type descriptions, command line menus, etc. [file 1471-2105-9-523-S1.zip › USeqUserGuides/JavaDoc.zip!/JavaDoc/index-files/index-6.html]

 
 
 
 
 
 
F-Index
 


 

 
function windowTitle()
{
    parent.document.title="F-Index";
}
 
 
 

 

 


 
   
  
 
 
 
   
 
   
          Overview     
        Package   
        Class   
        Use   
          Tree     
          Deprecated     
       Index    
          Help     
   
 
 
  
 
 
 

 
  
   PREV LETTER   
   NEXT LETTER    
  
    FRAMES     
   NO FRAMES     
  
    All Classes  ');
  }
  //-->
 
 
    All Classes  
 


  
 
 
  
 

 A   B   C   D   E   F   G   H   I   J   L   M   N   O   P   Q   R   S   T   U   V   W   X   Z   
    
 F  
 
   factorial(int)   - 
Static method in class util.gen. Num 
 Calculates N! modified from http://www.unix.org.ua/orelly/java-ent/jnut/ch01_03.htm
 Good for n    factorialApproximation(double)   - 
Static method in class util.gen. Num 
 Srivivasa Ramanuja ln(n!) factorial estimation, better than Stirling's approximation.
   Fasta   - Class in  util.bio.parsers      Fasta(String, String)   - 
Constructor for class util.bio.parsers. Fasta 
  
   FDR_ID   - 
Static variable in class edu.utah.seq.analysis. ScanSeqs 
  
   FDRVsQValues   - Class in  trans.main      FDRVsQValues()   - 
Constructor for class trans.main. FDRVsQValues 
  
   FDRWindowConverter   - Class in  trans.main  Associates an empirical FDR estimate with each window provided a mock IP was performed.   FDRWindowConverter(String[])   - 
Constructor for class trans.main. FDRWindowConverter 
  
   Feature   - Class in  bioroot      Feature(String)   - 
Constructor for class bioroot. Feature 
  
   fetchAllCenters(String)   - 
Method in class util.bio.seq. OligoTiler 
  
   fetchAllFilesRecursively(File)   - 
Static method in class util.gen. IO 
 Fetches files that don't start with a '.' from a directory recursing through sub directories.
   fetchAllFilesRecursively(File, String)   - 
Static method in class util.gen. IO 
 Fetches all files with a given extension in a directory recursing through sub directories.
   fetchAllS3ObjectKeyNames()   - 
Method in class util.amazon. S3Manager 
 Fetches the key names of the S3 objects.
   fetchAndAssignGATAParams(GATAParams)   - 
Method in class gata.main. PlotterPreferences 
  
   fetchAntibodyBeans(String, DBUtil)   - 
Static method in class bioroot.antibody. AntibodySpreadSheet 
 Build AntibodyBean[] from Antibody table in database.
   fetchAntibodyTableRows(UserBean, AntibodyBean[], int[], DBUtil, boolean, boolean)   - 
Static method in class bioroot.antibody. AntibodySpreadSheet 
 Returns table rows for every oligo according to a Preference, if allTabs==true, then all features are
 returned in a tab delimited format, otherwise its HTML.
   fetchArrayList(File)   - 
Static method in class gata.main. GATAUtil 
  
   fetchArrayList(File)   - 
Static method in class util.gen. IO 
 Fetches an ArrayList stored as a serialize object file.
   fetchArrowButton(String, String, String, int)   - 
Static method in class util.gen. HTML 
 Returns an upDown arrow ala Mac look and feel that will set a formParm to a given value and
 submit the form provided the form is named "form" (ie  ).
   fetchBLASTParams(int, int)   - 
Static method in class gata.main. GATAUtil 
 Returns Lambda and K or null for a given match and misMatch from BLASTN.
   fetchBLASTParams(int, int)   - 
Static method in class util.bio.calc. Alignment 
 Returns Lambda, K, H or null for a given match and misMatch from BLASTN.
   fetchBoolean(String)   - 
Method in class trans.main. T2Parameter 
  
   fetchBottomCenterY(Cluster)   - 
Method in class util.bio.cluster. ClusterDrawPanel 
 Recurse through clusters always going lower to find center
   fetchCelFileStatsFiles()   - 
Method in class trans.qc. CelFileQualityControl 
  
   fetchCellTypeConcats(int, UserBean)   - 
Method in class bioroot. DBUtil 
 Fetches concatinate of name and notes from CellType
   fetchCenteredFowardAndReverseCounts()   - 
Method in class util.gen. Histogram 
 Returns two columns as int[columns][counts] centered on max value with zeros as filler.
   fetchCheckBoxNumberTableRows(Feature[], int)   - 
Static method in class bioroot. Feature 
  
   fetchCheckGroup(String, String, String[])   - 
Static method in class util.gen. HTML 
 Returns a selected check box group with the toSelect selected if present in the String[]
   fetchChromosomeFiles(File[], String)   - 
Static method in class trans.cel. MakeChromosomeSets 
 Extracts all the Files with a given chromosome name from a list of directories
   fetchChromosomeSpecificBRs(BindingRegion[], String)   - 
Static method in class trans.anno. IntersectRegions 
 Given a sorted array of BindingRegion and a chromosome, returns the appropriate BindingRegions.
   fetchChromSpecificWindowScores(String)   - 
Method in class edu.utah.seq.analysis. QCSeqs 
  
   fetchChromStrandName()   - 
Method in class edu.utah.seq.parsers. ElandExportLine 
  
   fetchClosestOligoScore(Oligo[], int)   - 
Static method in class trans.graphics. IntervalDrawPanel 
 Finds the closes Oligo to a bp point and returns the indexed score.
   fetchClusterCenterY(Cluster)   - 
Method in class util.bio.cluster. ClusterDrawPanel 
 Returns the 'middle' Y coordinate for the cluster.
   fetchColumn(String)   - 
Method in class bioroot. DBUtil 
 Returns a String[] of the items in a particular column.
   fetchConcatMakeIdLast(String, int, String, int)   - 
Method in class bioroot. DBUtil 
 Returns a concatinate of the results where each String in the String[] represents a results
 table row where each column entry is separated by the divider String.
   fetchConcatMakeIdLast(String, int, String, int)   - 
Method in class util.gen. SQL 
 Returns a concatinate of the results where each String in the String[] represents a results
 table row where each column entry is separated by the divider String.
   fetchConcentrationGroup(OligoBean)   - 
Static method in class bioroot.oligo. OligoBase 
 Returns a concentration unit
   fetchControlIntensities(Quantile[])   - 
Method in class trans.cel. QuantileNormalization 
 Uses the control indexes to fetch the control intensities from the Quantile[].
   fetchControls()   - 
Method in class edu.utah.seq.analysis. DefinedRegionScanSeqs 
 Fetch control Data for each directory, will merge strands if needed.
   fetchControls()   - 
Method in class edu.utah.seq.analysis. ScanSeqs 
 Fetch control Data for each directory, will merge strands if needed.
   fetchExit(String, HashMap)   - 
Static method in class util.gen. Misc 
 Looks for key, if not found prints error message and exists.
   fetchFalseBlocks(boolean[], int, int)   - 
Static method in class util.bio.annotation. ExportIntergenicRegions 
  
   fetchFile(String)   - 
Method in class trans.main. T2Parameter 
  
   fetchFileNames(File[])   - 
Static method in class util.gen. IO 
 Returns the names of the files or directories.
   fetchFiles(PlasmidBean[], StrainBean[], AntibodyBean[], String, DBUtil)   - 
Static method in class bioroot. Archive 
 Fetches any plasmid or strain files.
   fetchFiles(String[], String)   - 
Static method in class util.gen. IO 
 For each base name (ie /home/data/treat1 ), makes a File object using the chromExtenstion
 (ie /home/data/treat1.chromExtension ), if it actually exists, it is saved and a File[]
 returned.
   fetchFilesRecursively(File)   - 
Static method in class util.gen. IO 
 Fetches all files in a directory recursing through sub directories.
   fetchFilesRecursively(File, String)   - 
Static method in class util.gen. IO 
 Fetches all files with a given extension in a directory recursing through sub directories.
   fetchFloatArrays(String, boolean)   - 
Static method in class trans.main. ScanChip 
 Given a directory (or a file), fetches serialized float[] from all files with the .celp extension.
   fetchFloatFloatArrays(File[])   - 
Static method in class util.gen. IO 
 Loads float[][]s from disk.
   fetchGCContent(Region[])   - 
Method in class trans.anno. RandomRegions 
 Converts the Region[] sequences into boolean[]s, everything not g or c are recorded as false.
   fetchGCContent(char[])   - 
Static method in class util.bio.seq. Seq 
 Converts a DNA sequence into a boolean[], everything not g or c is recorded as false.
   fetchGeneConcat(int, UserBean)   - 
Method in class bioroot. DBUtil 
 Fetches concatinate of name and notes from Gene
   fetchGeneNames(int)   - 
Method in class bioroot. DBUtil 
 Fetches geneNames given a labGroupId, assumes connection to BioRoot+Database.
   FetchGenomicSequences   - Class in  util.bio.parsers  Fetches the genomic sequences for a list of regions.   FetchGenomicSequences(String[])   - 
Constructor for class util.bio.parsers. FetchGenomicSequences 
  
   fetchGoodWindow()   - 
Method in class edu.utah.seq.analysis. EnrichedRegionMaker 
  
   fetchGoodWindow()   - 
Method in class trans.main. IntervalMaker 
  
   fetchGoodWindow()   - 
Method in class trans.main. MultiWindowIntervalMaker 
  
   fetchGoodWindow(double)   - 
Method in class trans.main. SetNumberIntervalMaker 
  
   fetchHTMLTableRow()   - 
Method in class bioroot. CellTypeBean 
  
   fetchHTMLTableRow()   - 
Method in class bioroot. GeneBean 
  
   fetchHTMLTableRow()   - 
Method in class bioroot. MarkerBean 
  
   fetchHTMLTableRow()   - 
Method in class bioroot. OrganismBean 
  
   fetchIdNameMatchArray(String)   - 
Method in class bioroot. DBUtil 
 Fetches an IndexNameMatch Array.
   FetchIndexedSequence   - Class in  util.bio.seq  Fetches sequence given a directory of indexed sequences, see IndexFastas.   FetchIndexedSequence(File, boolean)   - 
Constructor for class util.bio.seq. FetchIndexedSequence 
  
   fetchIntensities(File[], String)   - 
Static method in class trans.main. MultiSGRPrinter 
 Fetches a float[] from each of the directories given a fileName.
   fetchIntensities(File[], String, boolean)   - 
Static method in class trans.main. ScanChromosomes 
 Fetches a float[] from each of the directories given a fileName.
   fetchIntensities(File[], String, boolean)   - 
Static method in class trans.main. ScanChromosomesCNV 
 Fetches a float[] from each of the directories given a fileName.
   fetchIntensityFeatures(File[])   - 
Static method in class trans.cel. MakeChromosomeSets 
 Loads IntensityFeature[]s into a single master.
   fetchIntervalLine(Interval, int)   - 
Method in class trans.main. IntervalReportPrinter 
  
   fetchIntervalReport(Interval, int)   - 
Method in class trans.main. IntervalReportPrinter 
  
   fetchIntrons(ArrayList)   - 
Static method in class gata.main. GATAUtil 
 Takes an ArrayList of exon startStop int[2]'s, returns an ArrayList of int[2]'s representing
         the start,stop for each intron
   fetchIntrons(boolean[], UCSCGeneLine[])   - 
Method in class util.bio.annotation. ExportIntronicRegions 
  
   fetchIntrons(ArrayList)   - 
Static method in class util.bio.annotation. GeneGroup 
 Takes an ArrayList of exon startStop int[2]'s, returns an ArrayList of int[2]'s representing
         the start,stop for each intron
   fetchLabGroupName(int)   - 
Method in class bioroot. DBUtil 
 Fetches an labGroupName given an id, assumes connection to BioRoot+Database.
   fetchLabGroupNames()   - 
Method in class cgar. Util 
 Returns LabGroup.name, initiates an sql connection and the closes it so don't call if
 you are doing anything else.
   fetchLabGroups(String[])   - 
Method in class bioroot. DBUtil 
 Returns a sorted list of lab groups.
   fetchLoadedOligos(File)   - 
Static method in class trans.bkgrnd. CalcBkgrndRatios 
  
   fetchLoadedOligos(File)   - 
Static method in class trans.bkgrnd. CalculateBackgroundStats 
  
   fetchMarkerConcats(int)   - 
Method in class bioroot. DBUtil 
 Fetches concatinate of name and notes from Marker
   fetchMarkerNames()   - 
Method in class bioroot. DBUtil 
 Fetches markerNames given an labGroupId, assumes connection to BioRoot+Database.
   fetchMatchingStringArrayIndex(String, String[])   - 
Static method in class util.gen. Misc 
 Loose matching of substring guess from larger String[]s.
   fetchMatrixValues(float[][], int[][])   - 
Static method in class util.gen. Num 
 Given a square table and a list of coordinates, will return the associated values.
   fetchMaxOligoScore(Oligo[], int, int)   - 
Static method in class trans.graphics. IntervalDrawPanel 
 Scans oligos for a max score when given a bp range.
   fetchMedianQuantileValue(Quantile[])   - 
Static method in class trans.cel. QuantileNormalization 
 Calculates the median intensity of a sorted Quantile[].
   fetchMinScore(AlignParams[])   - 
Static method in class gata.plotter. MultiAlignPanel 
 Finds lowest minScore in an array of AlignParams.
   fetchMultipleCells(String)   - 
Method in class bioroot. DBUtil 
 Returns a String[] of the results.
   fetchMultipleCells(String)   - 
Method in class util.gen. SQL 
 Returns a String[] of the results.
   fetchMultipleOptionGroup(String[], String[])   - 
Static method in class util.gen. HTML 
 Returns a selected option group with the toSelect selected if present in the String[]
 of options.
   fetchNames()   - 
Method in class bioroot. Preference 
 returns a String[] of names
   fetchNamesAndDirectories(File)   - 
Static method in class util.gen. IO 
 Given a directory, returns a HashMap of the containing directories' names and a File obj for the directory.
   fetchNewRegions(int, int, int, int)   - 
Method in class gata.plotter. ConservedSeqs 
  
   fetchNumberedFiles(String, File)   - 
Method in class trans.main. T2Parameter 
  
   fetchNumberedOneFile(String, File)   - 
Method in class trans.main. T2Parameter 
  
   fetchObject(File)   - 
Static method in class gata.main. GATAUtil 
  
   fetchObject(File)   - 
Static method in class util.gen. IO 
 Fetches an Object stored as a serialized file.
   fetchOligoBeans(String, DBUtil)   - 
Static method in class bioroot.oligo. OligoSpreadSheet 
 Build OligoBean[] from Oligo table in database.
   fetchOligoTableRows(Preference, OligoBean[], int[], DBUtil, boolean, boolean)   - 
Static method in class bioroot.oligo. OligoSpreadSheet 
 Returns table rows for every oligo according to an OligoPreference, if allTabs==true, then all features are
 returned in a tab delimited format, otherwise its HTML.
   fetchOptionGroup(String, String[])   - 
Static method in class util.gen. HTML 
 Returns a selected option group with the toSelect selected if exactly matches one of the String[] options
   fetchOptionGroupLoose(String, String[])   - 
Static method in class util.gen. HTML 
 Returns a selected option group with the toSelect selected if at all present in the String[] options.
   fetchOrganismConcat(int)   - 
Method in class bioroot. DBUtil 
 Fetches concatinate of name and notes from Organism
   fetchPermutatedArrays()   - 
Method in class trans.main. RandomizeLabels 
 Attempts to fetch a unique, unseen permutation of the t[][] and control[][] arrays.
   fetchPlasmidBeans(String, DBUtil)   - 
Static method in class bioroot.plasmid. PlasmidSpreadSheet 
 Build PlasmidBean[] from Plasmid table in database.
   fetchPlasmidTableRows(UserBean, PlasmidBean[], int[], DBUtil, boolean, boolean)   - 
Static method in class bioroot.plasmid. PlasmidSpreadSheet 
 Returns table rows for every oligo according to a Preference, if allTabs==true, then all features are
 returned in a tab delimited format, otherwise its HTML.
   fetchPoint()   - 
Method in class edu.utah.seq.parsers. ElandExportLine 
  
   fetchPointData(File)   - 
Method in class edu.utah.seq.data. PointData 
 Loads a directory of xxx.bar PointData files into a HashMap where the
 key =
   fetchPointData(File[], File[], boolean)   - 
Static method in class edu.utah.seq.data. PointData 
 Multi directory version of fetchPointData(), set mergeDirectories to null to keep everything loaded in memory.
   fetchPointData(File, File, boolean)   - 
Static method in class edu.utah.seq.data. PointData 
 Fetches PointData from a directory.
   fetchPossibleValue(String)   - 
Method in class trans.main. T2Parameter 
  
   fetchProximalWindows(GeneGroup[], RocWindow[], int)   - 
Static method in class trans.roc. ParsePatternedWindows 
  
   fetchQCParams()   - 
Method in class trans.qc. CelFileQualityControl 
  
   fetchRadio(String, String, String)   - 
Static method in class util.gen. HTML 
 Returns a radio input line with CHECKED if the value is equal to the potential
   fetchRadioGroup(String, String, String[])   - 
Static method in class util.gen. HTML 
 Returns a selected radio group with the toSelect selected if present in the String[]
   fetchRadioGroupSubmit(String, String, String[], String)   - 
Static method in class util.gen. HTML 
 Returns a selected radio group with the toSelect selected if present in the String[]
 Also throws in a little javascript to submit the form if the button is clicked.
   fetchRandomCoordinates(int, int)   - 
Method in class trans.anno. RandomRegions 
 Given a desired length of sequence, returns multiple random region start stop coordinates.
   fetchRandomCoordinates(int, int, double)   - 
Method in class trans.anno. RandomRegions 
 Given a desired length of sequence, returns multiple random region start stop coordinates.
   fetchRandomObservations(HashMap<String, PointData>, int)   - 
Static method in class edu.utah.seq.data. PointData 
 Lots of work to fetch random observation.
   fetchReaderOnZippedFile(File)   - 
Static method in class util.gen. IO 
 Returns a BufferedReader from which you can call readLine() directly from a zipped file without decompressing.
   fetchReadLength()   - 
Method in class edu.utah.seq.parsers. ElandExportLine 
  
   fetchReagentCounts(UserBean, String)   - 
Method in class bioroot. DBUtil 
 Fetches the number of reagents in the users labgroup, and the number owned by the user.
   fetchReagentNames(int, String)   - 
Method in class bioroot. DBUtil 
 Fetches the sorted reagent names associated with a labGroup.
   fetchRecentReagentConcats(int, String)   - 
Method in class bioroot. DBUtil 
 Fetches concatinate of name and notes from Plasmids
   fetchRegionGrs(String, int, int)   - 
Method in class trans.roc. ScoreParsedBars 
 Fetches the Grs under the region,
 Uses the bpPositionOffSets so set appropriately!
   fetchRenderingHints()   - 
Static method in class gata.main. GATAUtil 
 Map of hints for high quality/ slow rendering
   fetchRenderingHints()   - 
Static method in class util.gen. Swing 
 Map of hints for high quality/ slow rendering
   fetchResultConcat(String, int, String, int)   - 
Method in class bioroot. DBUtil 
 Returns a concatinate of the results where each String in the String[] represents a results
 table row where each column entry is separated by the divider String.
   fetchResultConcat(String, int, String, int)   - 
Method in class util.gen. SQL 
 Returns a concatinate of the results where each String in the String[] represents a results
 table row where each column entry is separated by the divider String.
   fetchSameNumberLines(BufferedReader, int)   - 
Method in class trans.tpmap. MummerMapper 
 Loads the mummer results for one oligo into an ArrayList.
   fetchSearchColumns()   - 
Method in class bioroot. SearchBean 
 Extracts which columns to search for given group selections and field selections
   fetchSeq(String)   - 
Static method in class gata.plotter. ConservedSeqs 
  
   FetchSeqsQualsByName   - Class in  util.bio.parsers      FetchSeqsQualsByName(String[])   - 
Constructor for class util.bio.parsers. FetchSeqsQualsByName 
  
   fetchSequence(String, int, int)   - 
Method in class util.bio.seq. FetchIndexedSequence 
 Returns null if chromsome doesn't exist or the start or stop indexedSequence out of range.
   fetchSequences()   - 
Method in class gata.plotter. ConservedSeqs 
  
   fetchSequences(int[][])   - 
Method in class util.bio.seq. FetchIndexedSequence 
 Assumes startStop[index][0 start, 1 stop] is sorted by starts.
   fetchSequences(String, int[][])   - 
Method in class util.bio.seq. FetchIndexedSequence 
 Returns null if chromsome doesn't exist or the start or stop indexedSequence out of range.
   fetchSequences(String[])   - 
Static method in class util.bio.xamer. XamerCounter 
 Fetch all sequences from each multi-FASTA file
   fetchSingleColumn(String, String)   - 
Method in class bioroot. DBUtil 
 Returns a String[] of the items in a particular column.
   fetchSingleColumn(String)   - 
Method in class bioroot. DBUtil 
 Returns a int[] of the items in the first column.
   fetchSingleColumn(String, String)   - 
Method in class util.gen. SQL 
 Returns a String[] of the items in a particular column.
   fetchStrainBeans(String, DBUtil)   - 
Static method in class bioroot.strain. StrainSpreadSheet 
 Build StrainBean[] from Strain table in database.
   fetchStrainTableRows(UserBean, StrainBean[], int[], DBUtil, boolean, boolean)   - 
Static method in class bioroot.strain. StrainSpreadSheet 
 Returns table rows for every oligo according to a Preference, if allTabs==true, then all features are
 returned in a tab delimited format, otherwise its HTML.
   fetchStrandedPointData(File[])   - 
Static method in class edu.utah.seq.data. PointData 
 Multi directory version of fetchStrandedPointData().
   fetchStrandedPointData(File)   - 
Static method in class edu.utah.seq.data. PointData 
 Fetches, but doesn't load or merge, PointData, returning a chromosome split hashmap.
   fetchSubSequence(int, int, int, String)   - 
Static method in class util.bio.seq. Seq 
 Returns a sub sequence given a relative start and stop, the bp for the first base.
   fetchSubSequenceInterbaseCoordinates(int, int, int, String)   - 
Static method in class util.bio.seq. Seq 
 Returns a sub sequence given a relative start and stop, the bp for the first base.
   fetchSumaryLine()   - 
Method in class util.bio.annotation. GeneGroup 
  
   fetchTabbedTableColumnRow(Feature[])   - 
Static method in class bioroot. Archive 
 Returns a table header row using the Preferece obj
   fetchTableColumnRow(Preference, String)   - 
Static method in class bioroot. Util 
 Returns a table header row using the Preferece obj and a reagentType: Oligo, Plasmid, Strain...
   fetchTextCelFileIntensities(File)   - 
Static method in class trans.misc. Util 
 Fetches intensity values from a text version cel file.
   fetchTimedURLs(int, String[])   - 
Method in class util.amazon. S3Manager 
 Returns URLs to download particular objects from S3 that expire after a given amount of time.
   fetchTopCenterY(Cluster)   - 
Method in class util.bio.cluster. ClusterDrawPanel 
 Recurse through clusters always going higher to find center
   fetchUserBean(HttpServletRequest, HttpServletResponse)   - 
Static method in class bioroot. Util 
 Sets a guest user bean if there is none.
   fetchUserName(int, boolean)   - 
Method in class bioroot. DBUtil 
 Returns firstName lastName or lastName, firstName given a user id number
   fetchValue(String)   - 
Method in class trans.main. T2Parameter 
  
   fetchValueOptionGroup(String, String[])   - 
Static method in class util.gen. HTML 
 Returns a selected option group with the toSelect selected if present in the String[].
   FileCrossFilter   - Class in  expr      FileCrossFilter(String[])   - 
Constructor for class expr. FileCrossFilter 
  
   FileFocusListener   - Class in  gata.aligner      FileFocusListener()   - 
Constructor for class gata.aligner. FileFocusListener 
  
   FileInputForm   - Class in  gata.menu      FileInputForm(GATAParams)   - 
Constructor for class gata.menu. FileInputForm 
  
   FileInputPanel   - Class in  gata.menu      FileInputPanel(GATAParams, JFrame)   - 
Constructor for class gata.menu. FileInputPanel 
  
   FileJoiner   - Class in  util.apps  Joins text files into a single file, avoiding line concatenations.   FileJoiner(String[])   - 
Constructor for class util.apps. FileJoiner 
  
   FileMatchJoiner   - Class in  expr      FileMatchJoiner(String[])   - 
Constructor for class expr. FileMatchJoiner 
  
   filepath   - 
Static variable in class bioroot. Util 
  
   FileSize   - Class in  util.gen      FileSize(File)   - 
Constructor for class util.gen. FileSize 
  
   FileSplitter   - Class in  util.apps  Application for splitting a large text file into smaller files given a maximum number of lines.   FileSplitter(String[])   - 
Constructor for class util.apps. FileSplitter 
  
   FileTrimmer   - Class in  util.apps  Removes empty lines from text files in a given directory.   FileTrimmer(String[])   - 
Constructor for class util.apps. FileTrimmer 
  
   filter(boolean[], double)   - 
Method in class edu.utah.seq.data. PointData 
 Takes a boolean[] of masked/true positions and for every position, calculates the overlap of the read.
   filter(Window[])   - 
Method in class trans.main. MergeWindowArrays 
  
   filter(int, Positive[])   - 
Static method in class trans.roc. Positive 
 Adds the bpOffSetEnds to the end of each Positive.
   FilterAgilentProbeFile   - Class in  util.apps  Assumes one chromosome.   FilterAgilentProbeFile()   - 
Constructor for class util.apps. FilterAgilentProbeFile 
  
   filterAndSave(String)   - 
Method in class edu.utah.seq.data. FilterPointData 
 Removes PointData reads that overlap the regions.
   filterByStndDev()   - 
Method in class trans.main. IntervalFilter 
  
   filterChromosomeSpecificFeatures(ArrayList, ArrayList)   - 
Method in class trans.tpmap. TPMapProcessor 
  
   filterDNASeqLeaveWS(String)   - 
Static method in class util.bio.seq. Seq 
 Deletes any non IUP characters but leaves whitespaces
   filterDNASequence(String)   - 
Static method in class util.bio.seq. Seq 
 Deletes any non IUP characters
   filterDNASequenceStrict(String)   - 
Static method in class util.bio.seq. Seq 
 Deletes any non GATCNX characters
   filterField(String, int)   - 
Static method in class util.gen. HTML 
 Filters a String for bad characters replacing them with html equivalents.
   filterFields(String[], int)   - 
Static method in class util.gen. HTML 
 Filters a String[] for bad characters replacing them with html equivalents.
   filterGeneGroups(GeneGroup[], HashSet)   - 
Static method in class trans.anno. AnnotateRegions 
 Returns an array of GeneGroup whose names were found in the Hash
   filterGenes()   - 
Method in class expr. CorrelationMaps 
  
   filterIntervals()   - 
Method in class trans.main. T2 
 Creates set number of intervals.
   filterMapFeatures()   - 
Method in class trans.tpmap. TPMapProcessor 
  
   FilterPointData   - Class in  edu.utah.seq.data  Filters PointData for those that intersect a list of regions, e.g.   FilterPointData(String[])   - 
Constructor for class edu.utah.seq.data. FilterPointData 
  
   filterRocWindows(RocWindow[], double, boolean)   - 
Static method in class trans.roc. ParsePatternedWindows 
 Scans an array of RocWindow for those that are greater than (or less than if boolean is false) or
 equal to the cutOff.
   filterSubWindow()   - 
Method in class trans.main. IntervalFilter 
 Filters by the median ratio of the sub window
   filterTPMap()   - 
Method in class trans.tpmap. TPMapDuplicateFilter 
  
   FilterTPMapByRegions   - Class in  trans.tpmap  Filters a TPMap file by regions (tab delimited: chr start stop).   FilterTPMapByRegions(String[])   - 
Constructor for class trans.tpmap. FilterTPMapByRegions 
  
   filterWindows()   - 
Method in class edu.utah.seq.analysis. EnrichedRegionMaker 
  
   filterWindows(SmoothingWindow[])   - 
Method in class edu.utah.seq.data. HeatMapMakerPosNeg 
 Tosses any windows within the min max filter range.
   filterWindows(Window[])   - 
Method in class trans.main. WindowBlockMakerTwoColor 
 Tosses any windows within the min max filter range.
   filterWindowsByRegions()   - 
Method in class trans.main. IntervalMaker 
  
   filterWindowScore()   - 
Method in class trans.main. IntervalFilter 
  
   find(HashSet, String)   - 
Method in class util.bio.parsers. RefSeqAnnotationParser 
  
   findBestPair()   - 
Method in class util.bio.cluster. HierarchicalClustering 
 Takes an ArrayList of VCluster and finds the best pair based on a 
 PearsonCorrelationCoefficient.
   findBiggestGenomicRegion(GenomicRegion[])   - 
Static method in class trans.graphics. GenomicRegion 
 Returns the length of the biggest GenomicRegion
   findBin(Sgr)   - 
Method in class trans.roc. MethodTester 
 Finds an appropriate bin given an sgr object
   findBin(Sgr)   - 
Method in class trans.roc. SgrBacCounter 
 Finds an appropriate bin given an sgr object
   findBinHits(double)   - 
Method in class util.gen. Histogram 
 Given a value, finds the corresponding bin and returns the number of hits to that bin.
   findBinIndex(double)   - 
Method in class util.gen. Histogram 
 Returns the index of the containing bin or -1 if value is too small or big.
   findCenters(StartStop)   - 
Method in class util.bio.seq. OligoTiler 
  
   findChromPairs()   - 
Method in class trans.main. T2 
 Attempts to join smallest chromosomes together so long as the combine file doesn't exceed the size of the biggest.
   findClosestEndIndex(int[], int)   - 
Static method in class util.gen. Num 
 Finds the index of the key in the int[] but wont exceed the int[index]
   findClosestIndexToValue(int[], int)   - 
Static method in class util.gen. Num 
 Returns the index of the closest values[index] to the key.
   findClosestIndexToValue(float[], float)   - 
Static method in class util.gen. Num 
 Returns the index of the closest values[index] to the key.
   findClosestPeak(BindingRegion[], BindingRegion[])   - 
Static method in class trans.anno. IntersectBindingPeaks 
  
   findClosestPeaks(Interval[], Interval[], File, File)   - 
Method in class trans.main. OverlapCounter 
  
   findClosestStartIndex(int[], int)   - 
Static method in class util.gen. Num 
 Finds the index of the key in the int[] but wont preceed the int[index]
   findCommonAmplicons(Sample, Sample)   - 
Static method in class edu.utah.seq.methylation454. StatMethylationData 
  
   findCommonCpGs(Amplicon, Amplicon)   - 
Static method in class edu.utah.seq.methylation454. StatMethylationData 
  
   findCpGs(String)   - 
Static method in class edu.utah.seq.methylation454. Reference 
 Case sensitive.
   findDistanceToATG(BindingRegion, GeneGroup)   - 
Method in class trans.anno. AnnotateRegions 
 Finds the distance to conservative estimate of an ATG, returns 0 if overlaps.
   findDistanceToATG(BindingRegion, GeneGroup)   - 
Method in class trans.anno. AnnotateRegionsWithGeneList 
 Finds the distance to conservative estimate of an ATG, returns 0 if overlaps.
   findDistToClosestATG(BindingRegion)   - 
Method in class trans.anno. AnnotateRegions 
 Finds the distance to the closest ATG translation start site.
   findDistToClosestATG(BindingRegion, ArrayList)   - 
Method in class trans.anno. AnnotateRegions 
  
   findDistToClosestATG(BindingRegion)   - 
Method in class trans.anno. AnnotateRegionsWithGeneList 
 Finds the distance to the closest ATG translation start site.
   findDistToClosestATG(BindingRegion, ArrayList)   - 
Method in class trans.anno. AnnotateRegionsWithGeneList 
  
   findDistToClosestTranscript(BindingRegion)   - 
Method in class trans.anno. AnnotateRegions 
 Finds the distance to the closest ATG translation start site.
   findDistToClosestTranscript(BindingRegion, ArrayList)   - 
Method in class trans.anno. AnnotateRegions 
  
   findDistToClosestTranscript(BindingRegion, GeneGroup)   - 
Method in class trans.anno. AnnotateRegions 
 Finds the distance to conservative estimate of start of first exon, returns 0 if overlaps.
   findDistToClosestTranscript(BindingRegion)   - 
Method in class trans.anno. AnnotateRegionsWithGeneList 
 Finds the distance to the closest ATG translation start site.
   findDistToClosestTranscript(BindingRegion, ArrayList)   - 
Method in class trans.anno. AnnotateRegionsWithGeneList 
  
   findDistToClosestTranscript(BindingRegion, GeneGroup)   - 
Method in class trans.anno. AnnotateRegionsWithGeneList 
 Finds the distance to conservative estimate of start of first exon, returns 0 if overlaps.
   findEnds()   - 
Method in class selex. SeqRead 
  
   findExpressedGenes()   - 
Method in class trans.misc. CalculateGeneExtensions 
 Find genes with no neighbors, replaces the UCSCGeneLine[] array in the genes HashMap.
   findFlatTopPeak()   - 
Method in class trans.main. PeakPicker 
  
   findHighestFloat(float[])   - 
Static method in class util.gen. Num 
 Finds and returns the biggest float in an float[].
   findHighestInt(int[])   - 
Static method in class gata.main. GATAUtil 
  
   findHighestInt(int[])   - 
Static method in class util.gen. Num 
 Finds and returns the biggest int in an int[].
   findHighestPeak(SmoothingWindow[], int)   - 
Static method in class edu.utah.seq.analysis. PeakPicker 
 Find the window with the largest score.
   findHighestScore()   - 
Method in class trans.main. PeakPicker 
  
   findHighestScoringWindow(int[][], double[], double[])   - 
Static method in class trans.main. FindSubBindingRegions 
 Returns a partially complete SubWindow.
   findIndexes(int, int)   - 
Method in class edu.utah.seq.data. PointData 
 Given a start bp (included) and stop bp (not included), returns start (included) and stop (not included) indexes.
   findIsolatedGenes()   - 
Method in class trans.misc. CalculateGeneExtensions 
 Find genes with no neighbors, replaces the UCSCGeneLine[] array in the genes HashMap.
   findLastBase(Coordinate[])   - 
Static method in class util.bio.annotation. Coordinate 
 Finds the last base.
   findLowestScoringSeq(String[])   - 
Method in class meme. MotifScanner 
 Returns the lowest score from scoring all the seqs.
   findMatch(IdNameMatch[], String)   - 
Static method in class bioroot. IdNameMatch 
  
   findMaxIntIndex(int[])   - 
Static method in class util.gen. Num 
 Returns the first index containing the maximum value.
   findMinMaxDoubleValues(double[])   - 
Static method in class util.gen. Num 
 Finds min and max values of a float array.
   findMinMaxFloatArrays(float[][])   - 
Static method in class util.gen. Num 
 Finds min and max values of a float array.
   findMinMaxFloatValues(float[])   - 
Static method in class util.gen. Num 
 Finds min and max values of an unsorted float array.
   findMinMaxIntInt(int[][])   - 
Static method in class util.gen. Num 
 Finds min and max values of a int array.
   findMinMaxIntValues(int[])   - 
Static method in class util.gen. Num 
 Finds min and max values of a int array.
   findMinMaxPosition(PointData[])   - 
Static method in class edu.utah.seq.data. PointData 
 Finds the min and maximum positions from all of the position arrays.
   FindNeighboringGenes   - Class in  trans.anno  Finds closest genes and all genes within a given neighborhood.   FindNeighboringGenes(String[])   - 
Constructor for class trans.anno. FindNeighboringGenes 
  
   findOligoMatches(UserBean, PlasmidBean[], DBUtil)   - 
Static method in class bioroot.plasmid. PlasmidBase 
 Uses Blast to find oligo matches to a given array of plasmid from all the oligos in a labgroup visible to the user.
   findOligos(UserBean, PlasmidBean, DBUtil)   - 
Static method in class bioroot.plasmid. PlasmidBase 
 Uses Blast to find oligo matches to a plasmid from all the oligos in a labgroup visible to the user.
   findOligos(UserBean, PlasmidBean[], DBUtil)   - 
Static method in class bioroot.plasmid. PlasmidBase 
 Uses Blast to find oligo matches to a given array of plasmid from all the oligos in a labgroup visible to the user.
   findPeak(Interval)   - 
Static method in class trans.main. OverlapCounter 
 Returns base position of highest scoring peak, 
 if no peak uses middle of sub window
 if no sub window uses middle of interval
   findPeak()   - 
Method in class trans.main. PeakPicker 
  
   findPeakBp(int)   - 
Method in class trans.main. PeakPicker 
  
   findPeaks()   - 
Method in class edu.utah.seq.analysis. PeakPicker 
 Finds the best peak within each loaded enriched region.
   findRandomRegion(int)   - 
Method in class trans.anno. RandomRegions 
 Attempt to find a random Region of the appropriate minimum length.
   findRandomSegment(Region, int)   - 
Method in class trans.anno. RandomRegions 
 Returns a random segment of the appropriate length within the region.
   findRegion(GenomicRegion[], Point2D)   - 
Method in class trans.graphics. RankedSetDrawPanel 
 Scans an array of GenomicRegions Rectangles to see if the Point is present, ie they clicked it.
   findSeqSizeAndNumberReads(File)   - 
Method in class edu.utah.seq.compression. CompressFasta 
  
   findSequenceHits(String[], String)   - 
Static method in class util.bio.xamer. XamerCounter 
 Looks for an exact match of the oligo to each of the seqs, returning the seqs that contain
 the oligo
   findStartStopIndexes(int[], int, int, boolean)   - 
Static method in class util.gen. Num 
 Finds the start and stop indexes given a sorted int[] of values and two values, one after the other, 
 close together, otherwise just use Arrays.binarySearch.
   findStartStopThresholds(double, double)   - 
Method in class trans.roc. RocWindowScanner 
  
   FindSubBindingRegions   - Class in  trans.main  Finds the best sub window and binding peaks (using the   PeakPicker  ) within each interval.   FindSubBindingRegions(String[])   - 
Constructor for class trans.main. FindSubBindingRegions 
  
   findSubBindingRegions()   - 
Method in class trans.main. T2 
 Creates set number of intervals.
   findTargetFDRThreshold(double, int)   - 
Method in class trans.roc. RocWindowScanner 
 Attempts to find a threshold that gives the target FDR, will run through maxCycles and return closest threshold if targetFDR is not reached.
   findWindow(Window[])   - 
Method in class trans.main. MultiWindowIntervalMaker 
  
   findZeros(float[])   - 
Static method in class util.gen. Num 
 Scans a float array for zeros, if found returns true, other wise returns false.
   fireMeme(String)   - 
Method in class meme. MemeParser 
  
   fireMeme()   - 
Method in class meme. MemeR 
  
   fireMemeParallel(String, String, String, String)   - 
Method in class meme. MemeParser 
 Fires meme using a shell script and qsub, checks every 15 sec to see if done, returns unparsed MEME output.
   fireQSub(String, String, File)   - 
Static method in class util.gen. IO 
 Fires qsub with the commands.
   fireQSub(String, String, String, boolean)   - 
Static method in class util.gen. IO 
 Fires qsub with the commands, will wait and return results and delete x.sh and the output files if you like.
   flag   - 
Static variable in class util.apps.gump. Gump 
  
   flagSegmentedGenes()   - 
Method in class trans.misc. CalculateGeneExtensions 
  
   Flash   - Class in  util.apps      Flash(String[])   - 
Constructor for class util.apps. Flash 
  
   floatArrayToString(float[], String)   - 
Static method in class util.gen. Misc 
 Returns a String separated by separator for each float[i].
   floatArrayToString(float[], String)   - 
Static method in class util.gen. Num 
 Converts to String using delimiter.
   focusGained(FocusEvent)   - 
Method in class gata.aligner. FileFocusListener 
  
   focusLost(FocusEvent)   - 
Method in class gata.aligner. FileFocusListener 
  
   FONT   - 
Static variable in class util.apps.gump. GumpApplicationPanel 
  
   formatFraction(int, int, int)   - 
Static method in class edu.utah.seq.parsers. ElandMultiParser 
 Returns number space (percent%)
   formatFraction(int, int, int)   - 
Static method in class edu.utah.seq.parsers. ElandSequenceParser 
 Returns number space (percent%)
   formatNumber(double, int)   - 
Static method in class util.gen. Num 
 Converts a double ddd.dddddddd to a user determined number of decimal places right of the .
   formatNumberOneFraction(double)   - 
Static method in class util.bio.calc. NucleicAcid 
 Takes a double returns a String with one fractional digit  9.54443 -> 9.5
   formatNumberOneFraction(double)   - 
Static method in class util.gen. Num 
 Converts a double ddd.dddddddd to sss.s
   formatPercentOneFraction(double)   - 
Static method in class util.bio.calc. NucleicAcid 
 Takes a double in percent returns a String with one fraction 0.85842 -> 85.8%
   formatPercentOneFraction(double)   - 
Static method in class util.gen. Num 
 Converts 0.345543 to 34.6%
   formattedInfo(GenomicRegion)   - 
Method in class trans.graphics. RankedSetDrawPanel 
  
   fractionC()   - 
Method in class edu.utah.seq.methylation454. CpG 
  
   fractionPositive(ArrayList, double, double)   - 
Static method in class trans.roc. MethodTester 
 Given an ArrayList of Double, calculates the fraction that is >= scoreCutOff.
   fractionPositive(ArrayList, double, double)   - 
Static method in class trans.roc. SgrBacCounter 
 Given an ArrayList of Double, calculates the fraction that is >= scoreCutOff.
   fractionTotalIntersection(GenomicRegion[])   - 
Method in class trans.graphics. RankedSetAnalysis 
 Returns the total fraction of intersection.
   fullPathToBlast   - 
Static variable in class bioroot. Util 
  
 
 


 
   
  
 
 
 
   
 
   
          Overview     
        Package   
        Class   
        Use   
          Tree     
          Deprecated     
       Index    
          Help     
   
 
 
  
 
 
 

 
  
   PREV LETTER   
   NEXT LETTER    
  
    FRAMES     
   NO FRAMES     
  
    All Classes  ');
  }
  //-->
 
 
    All Classes  
 


  
 
 
  
 

 A   B   C   D   E   F   G   H   I   J   L   M   N   O   P   Q   R   S   T   U   V   W   X   Z
